# Dialysis Adequacy and Quality of Life Among Haemodialysis Patients: Does Facility Type Matter? A Comparison Between Government and Private Centres in Klang Valley, Malaysia

**DOI:** 10.1155/ijne/7175945

**Published:** 2026-08-25

**Authors:** Voon Son Wong, Yong Xiang Tong, Pooi Pooi Leong, Li Lian Tay, Norazinizah Ahmad Miswan, Teck Han Ng, Waye Hann Kang

**Affiliations:** ^1^ Department of Medicine, Universiti Tunku Abdul Rahman, Kajang, Selangor, Malaysia, utar.edu.my; ^2^ Department of Preclinical Science, Universiti Tunku Abdul Rahman, Kajang, Selangor, Malaysia, utar.edu.my; ^3^ Department of Nephrology, Hospital Sultan Idris Shah Serdang, Kajang, Selangor, Malaysia; ^4^ Department of Medicine, Ampang Hospital, Ampang, Selangor, Malaysia, duhs.edu.pk

## Abstract

**Introduction:**

Patients with end‐stage kidney disease (ESKD) receiving haemodialysis (HD) face a substantial burden of physical and psychological challenges, with dialysis adequacy recognised as a key determinant of quality of life (QoL). The number of HD patients in Malaysia continues to grow, with more than half receiving treatment at private dialysis centres. This study compares dialysis adequacy and QoL between patients attending government and private HD centres in Klang Valley, Malaysia.

**Materials and Methods:**

An observational cross‐sectional study was conducted in three private and two government dialysis centres in Selangor, Malaysia, between August and December 2024. Data were collected from 294 HD patients using a structured questionnaire on sociodemographic and clinical information, alongside the KDQOL‐36. Good dialysis adequacy was defined a priori as single‐pool Kt/V > 1.2, with a stricter threshold of Kt/V > 1.5 examined as a sensitivity analysis.

**Results and Discussion:**

Private HD centre patients were significantly older (*p* < 0.001) but had lower dialysis vintage (*p* < 0.001), lower Kt/V (*p* < 0.001) and higher iPTH levels (*p* = 0.033). Government HD centre patients had significantly better physical functioning (*p* < 0.001) and SF‐12 physical scores (*p* = 0.019). At the primary threshold (Kt/V > 1.2), the independent predictors of good dialysis adequacy after multivariable adjustment were female gender (OR = 2.37, 95% CI 1.10–5.09, *p* = 0.027), treatment at a government centre (OR = 3.60, 95% CI 1.32–9.86, *p* = 0.013) and lower dry weight (OR = 0.96 per kg, 95% CI 0.93–0.98, *p* < 0.001). These predictors were unchanged in the sensitivity analysis at Kt/V > 1.5, in which higher educational attainment additionally predicted poorer adequacy. Dialysis adequacy was not independently associated with QoL; physical QoL was predicted by younger age and shorter dialysis vintage and mental QoL by female gender and Malay ethnicity.

**Conclusions:**

Government HD centre patients in Malaysia had significantly better dialysis adequacy than those in private centres. Female gender and government centre affiliation were independent predictors of good Kt/V, whereas higher dry weight was associated with poorer adequacy. Most KDQOL domains were comparable between facility types, though government centre patients reported better physical functioning and physical component scores. These findings highlight the need for regulatory oversight of dialysis practice standards across facility types and suggest that individualised prescription optimisation, particularly to patient body size, is an important target for improving dialysis adequacy in Malaysia.

## 1. Introduction

The number of patients living with end‐stage kidney disease (ESKD) in Malaysia is growing, with a prevalence of 0.36% of the total population reported in 2019 [1, 2]. This figure has risen substantially over the past two decades, placing considerable strain on healthcare delivery and resources. Haemodialysis (HD) is the most common form of renal replacement therapy in Malaysia, with 53.8% of patients receiving treatment at private dialysis centres, while the remainder attend government or non‐governmental not‐for‐profit facilities [3]. Funding these services is a particular challenge given Malaysia’s mixed public–private healthcare model, in which almost all ESKD patients receive government subsidies for their renal replacement therapy, regardless of whether they attend a public or private centre [2].

Beyond survival, maintaining an acceptable quality of life (QoL) is a central concern for patients on HD. The disease itself, combined with the demands of thrice‐weekly treatment sessions, imposes significant physical, psychological and social burdens—from fatigue and dietary restrictions to the disruption of employment and family life [4]. Several instruments have been developed to capture these dimensions of health‐related QoL in patients with kidney disease, of which the Kidney Disease Quality of Life (KDQOL) questionnaire is among the most widely used [5]. By examining disease‐specific domains including symptom burden, the effects of kidney disease on daily activities and dialysis staff support, the KDQOL enables clinicians to identify targeted areas for intervention, whether in symptom management, psychological support or social functioning [5, 6].

A key measure of HD effectiveness is dialysis adequacy, most commonly quantified using Kt/V:the product of dialyser clearance (K) and session duration (t), divided by the patient’s urea distribution volume (V) [7]. A higher Kt/V reflects more efficient urea removal and is associated with better survival, fewer hospitalisations and improved symptom control. Clinicians use Kt/V routinely to guide adjustments to dialysis prescriptions, making it a practical as well as a prognostic tool. Evidence suggests that the relationship between dialysis adequacy and QoL is complex and likely bidirectional: while better adequacy generally reduces symptom burden and supports physical functioning, poor QoL may itself impair adherence to the treatment schedule, in turn reducing measured Kt/V [8, 9].

Despite the growing burden of ESKD and the expanding role of private dialysis centres in Malaysia, data comparing dialysis adequacy and health‐related QoL between public and private HD facilities in this setting remain scarce. While national registry data provide aggregate Kt/V statistics, they do not disaggregate outcomes by facility type or examine the sociodemographic and clinical predictors that may differ systematically between the two sectors. This is a critical gap, given that Malaysia’s mixed public–private funding model, in which patients in both sectors receive government subsidies, and also creates a unique context not directly comparable to the healthcare systems examined in most international literature. Furthermore, although instruments, such as the KDQOL, have been validated for use in Malaysia, no published study has used it to compare QoL between government and private HD centres. Understanding whether dialysis adequacy and QoL differ between facility types, and what factors predict adequacy in this local context, is essential for informing targeted policy and clinical interventions. This study therefore aims to examine dialysis adequacy and QoL among HD patients across government and private centres in Klang Valley, Malaysia, and to identify their sociodemographic and clinical predictors.

## 2. Methodology

We conducted a cross‐sectional study between August and December 2024 across five dialysis centres in Selangor, Malaysia. Institutional ethical approval was obtained from the Universiti Tunku Abdul Rahman Scientific and Ethical Review Committee and the Malaysia National Medical Research Registry (ID‐24‐03123‐ART).

Three private and two government dialysis centres in Klang Valley, each having provided HD services for at least 1 year, were selected. Convenience sampling was employed, and all ESKD patients aged 18 years and above at the participating centres were eligible for recruitment if they had a minimum of 3 months of HD history and were not acutely or critically ill at the time of data collection. Written informed consent was obtained from all participants before enrolment, and the confidentiality of all personal and clinical information was maintained throughout the study. Using the formula *n* = ( *Z* *α*/2 + *Z* *β*)^2^ × 2*σ* ^2^/*d* ^2^, we calculated that a minimum of 137 participants per subgroup (274 in total) was required to adequately power this study, with *Z* *α*/2 = 1.96 (95% confidence interval), *Zβ* = 0.84, *σ* = 0.3 for the expected standard deviation of Kt/V based on previous literature and *d* = 0.2 for the minimum clinically important difference in Kt/V [10].

Sociodemographic and clinical data were collected using a semistructured questionnaire. Household income was classified using the Malaysian Department of Statistics categories (B40 [bottom 40%], M40 [middle 40%] and T20 [top 20%]), with participants self‐identifying the category to which they belonged. Health‐related QoL was assessed using the validated Malay version of KDQOL‐36 Version 1.3, obtained under licence from the Mapi Research Trust. The KDQOL‐36 questionnaire is a validated disease‐specific instrument that encompasses five subscales: physical component summary, mental component summary, burden of kidney disease, symptoms and problems of kidney disease and effects of kidney disease on daily life [11]. The questionnaire was administered by trained multilingual research assistants who provided verbal clarification in Malay, English or Mandarin/Cantonese as needed, ensuring consistent comprehension across the multi‐ethnic cohort. Higher scores indicate better health‐related QoL [11, 12]. The KDQOL‐36 has been previously adapted and validated for use in the Malaysian population [11, 13].

Dialysis adequacy was assessed as single‐pool Kt/V (spKt/V), calculated by formal urea kinetic modelling using the Daugirdas equation from pre‐ and postdialysis serum urea obtained during a mid‐week dialysis session, together with treatment time, ultrafiltration volume and postdialysis weight. Kt/V values were obtained from the participating centres’ routine records; the same blood‐based method, consistent with the standard of the Malaysian Dialysis and Transplant Registry, was used across all five centres. Good dialysis adequacy was defined a priori as spKt/V > 1.2, in line with the KDOQI minimum target for thrice‐weekly HD [14, 15], and Kt/V ≤ 1.2 was considered poor. To assess the robustness of the findings to this definition, a sensitivity analysis was performed using a stricter threshold of Kt/V > 1.5, reflecting the mean delivered Kt/V reported by the Malaysian Dialysis and Transplant Registry [16].

Data were analysed using SPSS Version 27.0 (SPSS Inc., Chicago, IL). There were no missing data among the variables included in the analyses; no imputation was required, and all 294 participants were included in every analysis. Continuous variables were assessed for normality using the Shapiro–Wilk test. Normally distributed variables are presented as mean ± standard deviation and were compared between groups using the independent‐samples *t*‐test. Nonnormally distributed variables, specifically serum intact parathyroid hormone (iPTH) and alkaline phosphatase (ALP), are presented as median with interquartile range (IQR) and were compared using the Mann–Whitney *U* test. Categorical data are presented as frequency and percentage, and associations were assessed using the chi‐square test or Fisher’s exact test where expected cell frequencies fell below five.

Multivariable logistic regression was used to identify independent predictors of good dialysis adequacy. Variables were entered into the model if they were associated with dialysis adequacy at *p* < 0.25 on univariate analysis (gender, ethnicity, education level, dialysis centre type, dry weight, serum calcium, serum phosphate and dialysis vintage), or were considered a priori sociodemographic confounders (occupation and income level). Multicollinearity among the sociodemographic predictors was assessed using the generalised variance inflation factor, which confirmed the absence of significant collinearity. Sparse categories were collapsed before modelling (occupation as employed versus not employed; income as B40 versus M40/T20; education as primary or below, secondary and tertiary). In addition, multivariable linear regression was used to identify independent predictors of the SF‐12 physical and mental component summary scores, with age, gender, ethnicity, centre type, haemoglobin, dry weight, dialysis adequacy, dialysis vintage, income group and diabetes status entered as candidate predictors; the relationship between adequacy and QoL was further examined using Pearson’s correlation between continuous Kt/V and the KDQOL domain scores. A *p* value of < 0.05 was considered statistically significant.

## 3. Results

A total of 294 patients were recruited, exceeding the prespecified minimum sample size of 274. The cohort had a mean age of 55.3 years and was predominantly female (54.8%), with the largest proportion aged between 30 and 39 years (36.4%). Three‐quarters (75.9%) fell within the B40 lower‐income bracket. The most common causes of ESKD were hypertension and/or diabetes mellitus, accounting for 70.4% of cases, and patients had been on HD for an average of 6.6 years (Tables 1 and 2).

**TABLE 1:** Sociodemographic and clinical parameters according to dialysis centre and dialysis adequacy (*n* = 294).

Variables	Total (*n* = 294)	Private (*n* = 168)	Government (*n* = 126)	*p* value	Poor dialysis adequacy (*n* = 50)	Good dialysis adequacy (*n* = 244)	*p* value
Age, years				0.438			0.963
< 20	1	0	1		0	1	
20–29	48	31	17		9	39	
30–39	107	62	45		18	89	
40–49	60	29	31		12	48	
50–59	76	44	32		11	65	
60–70	1	1	0		0	1	
≥ 70	1	1	0		0	1	
Gender				0.344			< 0.001 crude OR = 3.897 (1.997–7.604)
Male	133	80	53		36	97	
Female	161	88	73		14	147	
Ethnicity				< 0.001			0.863
Malay	196	135	61		35	161	
Chinese	65	11	54		9	56	
Indian	29	21	8		5	24	
Others	4	1	3		1	3	
Education				0.809			0.055
No formal education	12	8	4		2	10	
Primary	42	23	19		1	41	
Secondary	173	101	72		33	140	
Tertiary	67	36	31		14	173	
Occupation				0.002			0.371
Unemployed	111	66	45		13	98	
Self‐employed	27	11	16		5	22	
Employee	37	15	22		6	31	
Retired	115	76	39		25	90	
Student	4	0	4		1	3	
Income				0.298			0.904
B40	223	122	101		37	186	
M40	64	42	22		12	52	
T20	7	4	3		1	6	
Marital				0.014			0.127
Single	51	19	32		6	45	
Married	208	127	81		42	166	
Divorced	13	7	6		1	12	
Widowed	22	15	7		1	21	
Cause of ESKD				< 0.001			0.059
Diabetes	48	28	20		12	36	
Hypertension	88	44	44		13	75	
Glomerular diseases	29	7	22		5	24	
Unknown	20	7	13		1	19	
Others	38	23	15		2	36	
Diabetes and hypertension	71	59	12		17	54	
Dialysis adequacy				< 0.001 crude OR:6.679 (3.947–11.300)			
Good	185	32	77				
Poor	109	136	49				

*Note:* Poor dialysis adequacy: Kt/V ≤ 1.2; good dialysis adequacy: Kt/V > 1.2; income level classified using the Malaysian Department of Statistics categories (B40 [bottom 40%], M40 [middle 40%] and T20 [top 20%]).

Abbreviation: ESKD, end‐stage kidney disease.

**TABLE 2:** Clinical parameters and KDQOL scores according to dialysis centre and dialysis adequacy.

Variables	Total (*n* = 294)	Private (*n* = 168)	Government (*n* = 126)	*p* value	Kt/V poor (*n* = 50)	Kt/V good (*n* = 244)	*p* value
Age, years	55.3 ± 13.42	57.8 ± 11.96	52.1 ± 14.56	^∗^< 0.001	55.2 ± 12.20	55.4 ± 13.67	0.926
Duration of HD, years	6.6 ± 5.68	4.9 ± 4.27	8.8 ± 6.52	^∗^< 0.001	4.8 ± 3.88	6.9 ± 6.92	^∗^0.015
Kt/V	1.51 ± 0.340	1.39 ± 0.293	1.68 ± 0.438	^∗^< 0.001			
Dry weight, kg	63.1 ± 16.09	67.1 ± 16.12	57.7 ± 12.43	^∗^< 0.001	75.9 ± 20.11	60.5 ± 13.79	^∗^< 0.001
Haemoglobin, g/dL	10.2 ± 1.72	9.99 ± 1.56	10.5 ± 1.89	^∗^0.011	10.2 ± 1.74	10.2 ± 1.72	0.927
Calcium (corrected), mmol/L	2.21 ± 0.238	2.20 ± 0.230	2.22 ± 0.249	0.414	2.12 ± 0.230	2.23 ± 0.237	^∗^0.005
Phosphate, mmol/L	1.68 ± 0.482	1.70 ± 0.435	1.64 ± 0.534	0.312	1.83 ± 0.563	1.65 ± 0.457	^∗^0.015
ALP, IU/L	113.0 (86.25)	114.0 (84.75)	112.5 (87.75)	^∗∗^0.903	110.0 (60.75)	116.5 (87.25)	^∗∗^0.281
iPTH, pg/mL	48.25 (58.45)	49.85 (55.78)	42.0 (67.63)	^∗∗^ ^,∗^0.033	49.2 (41.55)	47.2 (59.22)	^∗∗^0.620
Symptom/problem list	80.3 ± 14.81	79.6 ± 14.86	81.3 ± 14.74	0.317	74.8 ± 17.178	81.4 ± 14.05	^∗^0.004
Effects of kidney disease	76.8 ± 19.80	75.9 ± 19.66	78.0 ± 19.99	0.373	69.7 ± 22.29	78.2 ± 18.97	^∗^0.005
Burden of kidney disease	45.4 ± 29.4	45.7 ± 28.97	45.1 ± 30.13	0.864	38.2 ± 27.85	46.9 ± 29.57	0.058
Work status	40.6 ± 32.09	42.6 ± 26.03	38.1 ± 38.69	0.264	42.0 ± 30.90	40.4 ± 32.39	0.744
Cognitive function	76.1 ± 20.61	77.7 ± 19.61	74.0 ± 21.75	0.121	73.1 ± 21.04	76.7 ± 20.51	0.250
Quality of social interaction	81.9 ± 19.30	83.7 ± 18.42	79.6 ± 20.24	0.073	81.2 ± 19.78	82.0 ± 19.24	0.777
Sexual function	83.2 ± 25.79 (n = 75)	83.4 ± 21.60	82.8 ± 30.91	0.919	81.2 ± 25.88 (n = 8)	83.4 ± 25.97 (n = 67)	0.826
Sleep	65.5 ± 20.27	64.3 ± 20.18	67.0 ± 20.38	0.267	61.3 ± 19.75	66.3 ± 20.31	0.109
Social support	76.5 ± 21.45	76.0 ± 23.41	77.1 ± 18.59	0.329	76.3 ± 23.59	76.5 ± 21.03	0.109
Dialysis staff encouragement	78.1 ± 20.3	76.3 ± 20.48	80.4 ± 19.90	0.093	73.8 ± 21.47	78.9 ± 19.98	0.099
Overall health	63.8 ± 19.18	63.6 ± 19.47	64.0 ± 18.85	0.854	59.6 ± 1916	64.7 ± 19.10	0.088
Patient satisfaction	74.3 ± 21.84	74.4 ± 21.65	74.2 ± 22.17	0.939	76.7 ± 20.76	73.8 ± 22.06	0.405
Physical functioning	58.0 ± 26.36	53.2 ± 25.91	64.6 ± 25.63	^∗^< 0.001	51.0 ± 26.03	595 ± 26.25	^∗^0.038
Role limitations–physical	33.7 ± 41.79	32.1 ± 40.78	35.7 ± 43.18	0.469	25.5 ± 36.94	35.3 ± 42.59	0.129
Pain	71.2 ± 26.72	69.8 ± 27.38	73.0 ± 25.80	0.314	67.8 ± 27.10	71.9 ± 26.64	0.320
General health	52.9 ± 20.79	53.4 ± 21.09	52.3 ± 20.45	0.636	47.6 ± 20.88	54.0 ± 20.64	^∗^0.047
Emotional well‐being	75.9 ± 18.11	77.4 ± 18.27	74.0 ± 17.78	0.112	75.6 ± 18.97	76.2 ± 17.96	0.565
Role limitations–emotional	65.8 ± 43.36	67.1 ± 43.16	64.0 ± 43.74	0.553	56.7 ± 43.77	67.6 ± 43.13	0.104
Social function	69.9 ± 27.08	68.1 ± 27.11	72.2 ± 27.00	0.195	60.2 ± 27.28	71.8 ± 26.67	^∗^0.006
Energy/fatigue	57.0 ± 19.66	56.3 ± 19.93	57.8 ± 19.34	0.513	52.6 ± 20.58	57.8 ± 19.39	0.086
SF‐12 physical	38.3 ± 9.24	37.2 ± 9.59	39.7 ± 8.58	^∗^0.019	36.16 ± 8.94	38.70 ± 9.28	0.076
SF‐12 mental	50.8 ± 9.85	51.3 ± 10.20	50.1 ± 9.36	0.143	48.65 ± 10.55	51.22 ± 9.66	0.094

*Note:* Data are presented as mean ± standard deviation unless otherwise indicated; continuous variables are compared using the independent‐samples *t*‐test; KT/V, dialysis adequacy; ALP, alkaline phosphatase; SF‐12, Short Form Survey 12.

Abbreviation: iPTH, intact PTH.

^∗^ *p* < 0.05.

^∗∗^Nonnormally distributed variables assessed using the Mann–Whitney *U* test and presented as median (interquartile range).

Patients at private dialysis centres were significantly older and had shorter dialysis vintage, lower Kt/V, higher dry weight and higher serum iPTH levels compared to those at government centres. Haemoglobin was also significantly lower in private centre patients (9.99 ± 1.56 vs 10.5 ± 1.89 g/dL, *p* = 0.011). There were no significant differences in most QoL parameters between facility types; however, government centre patients scored significantly higher on physical functioning (*p* < 0.001) and SF‐12 physical component summary (*p* = 0.019) (Table 2).

When patients were grouped by dialysis adequacy (Kt/V > 1.2), those with good adequacy had significantly lower dry weight (60.5 ± 13.79 vs 75.9 ± 20.11 kg, *p* < 0.001), longer dialysis vintage (6.9 ± 5.92 vs 4.8 ± 3.88 years, *p* = 0.015), higher serum calcium (2.23 ± 0.237 vs 2.12 ± 0.230 mmol/L, *p* = 0.005) and lower serum phosphate (1.65 ± 0.457 vs 1.83 ± 0.563 mmol/L, *p* = 0.015) than those with poor adequacy. Several quality‐of‐life domains were also significantly better in the good‐adequacy group, including symptoms and problems of kidney disease (81.4 ± 14.05 vs 74.8 ± 17.18, *p* = 0.004), effects of kidney disease (78.2 ± 18.97 vs 69.7 ± 22.29, *p* = 0.005), physical functioning (59.5 ± 26.25 vs 51.0 ± 26.03, *p* = 0.038), general health (54.0 ± 20.64 vs 47.6 ± 20.88, *p* = 0.047) and social function (71.8 ± 26.67 vs 60.2 ± 27.28, *p* = 0.006) (Table 2).

On univariate analysis, several sociodemographic and clinical factors were significantly associated with good dialysis adequacy (Kt/V > 1.2) (Table 3). Female patients were almost four times as likely to achieve good adequacy as male patients (crude OR = 3.90, 95% CI 2.00–7.60, *p* < 0.001), and patients at government centres had more than four times the odds of those at private centres (crude OR = 4.20, 95% CI 1.96–9.01, *p* < 0.001). Higher dry weight was associated with lower odds of good adequacy (crude OR = 0.94 per kg, 95% CI 0.92–0.96, *p* < 0.001), as was higher serum phosphate (crude OR = 0.47 per mmol/L, 95% CI 0.25–0.87, *p* = 0.016). In contrast, higher serum calcium (crude OR = 6.18, 95% CI 1.70–22.50, *p* = 0.006) and longer dialysis vintage (crude OR = 1.09 per year, 95% CI 1.02–1.17, *p* = 0.017) were associated with greater odds of good adequacy. Higher educational attainment was associated with lower odds of good adequacy, with secondary (OR = 0.25, 95% CI 0.07–0.85, *p* = 0.026) and tertiary education (OR = 0.22, 95% CI 0.06–0.82, *p* = 0.024) each showing significantly lower odds than primary education or below. Ethnicity, employment status and income group were not significantly associated with adequacy on univariate analysis.

**TABLE 3:** Univariate and multivariate logistic regression analysis of independent variables (*n* = 294).

Variable	Univariate		Multivariable–Kt/V > 1.2 (primary)		Multivariable–Kt/V > 1.5 (sensitivity)	
	OR (95% CI)	*p*	aOR (95% CI)	*p*	aOR (95% CI)	*p*
Female (vs male)	3.90 (2.00–7.60)	^∗^< 0.001	2.37 (1.10–5.09)	^∗^0.027	3.23 (1.70–6.12)	^∗^< 0.001
Chinese (vs Malay)	1.35 (0.61–2.99)	0.455	0.45 (0.15–1.35)	0.154	1.01 (0.45–2.23)	0.986
Indian/others (vs Malay)	0.98 (0.38–2.55)	0.964	1.09 (0.36–3.34)	0.876	1.79 (0.67–4.78)	0.243
Secondary (vs ≤ primary)	0.25 (0.07–0.85)	^∗^0.026	0.38 (0.10–1.40)	0.147	0.30 (0.14–0.67)	^∗^0.003
Tertiary (vs ≤ primary)	0.22 (0.06–0.82)	^∗^0.024	0.40 (0.09–1.78)	0.226	0.27 (0.09–0.77)	^∗^0.014
Government (vs private)	4.20 (1.96–9.01)	^∗^< 0.001	3.60 (1.32–9.86)	^∗^0.013	7.66 (3.62–16.20)	^∗^< 0.001
Employed (vs not employed)	0.98 (0.47–2.05)	0.965	1.18 (0.47–2.94)	0.725	1.07 (0.50–2.27)	0.867
Household income status: M40/T20 (vs B40)	0.89 (0.44–1.78)	0.737	1.26 (0.52–3.04)	0.613	0.65 (0.31–1.37)	0.258
Dry weight (per kg)	0.94 (0.92–0.96)	^∗^< 0.001	0.96 (0.93–0.98)	^∗^< 0.001	0.97 (0.95–0.99)	^∗^0.010
Serum calcium (per mmol/L)	6.18 (1.70–22.50)	^∗^0.006	3.59 (0.72–17.78)	0.117	2.38 (0.66–8.52)	0.184
Serum phosphate (per mmol/L)	0.47 (0.25–0.87)	^∗^0.016	0.60 (0.27–1.37)	0.225	1.17 (0.63–2.18)	0.615
Dialysis vintage (per year)	1.09 (1.02–1.17)	^∗^0.017	1.03 (0.95–1.12)	0.458	1.00 (0.94–1.05)	0.882

*Note:* Good dialysis adequacy defined a priori as single‐pool Kt/V > 1.2 (primary analysis); Kt/V > 1.5 presented as a sensitivity analysis; all odds ratios reported in the directly modelled direction; OR > 1 indicates higher odds of good adequacy; sparse categories were collapsed before modelling (occupation: employed vs not employed; income: B40 vs M40/T20; education: ≤ primary, secondary, tertiary); reference categories: male, Malay, ≤ primary education, private centre, not employed, B40; KT/V, dialysis adequacy; ALP, alkaline phosphatase; iPTH, intact parathyroid hormone.

Abbreviations: aOR, adjusted odds ratio; CI, confidence interval; OR, odds ratio.

^∗^ *p* < 0.05.

On multivariable logistic regression, three variables remained independent predictors of good dialysis adequacy: female gender (adjusted OR = 2.37, 95% CI 1.10–5.09, *p* = 0.027), treatment at a government centre (adjusted OR = 3.60, 95% CI 1.32–9.86, *p* = 0.013) and lower dry weight (adjusted OR = 0.96 per kg, 95% CI 0.93–0.98, *p* < 0.001). Education level, serum calcium, serum phosphate and dialysis vintage were no longer significant after adjustment, indicating that their univariate associations were attenuated by the other predictors (Table 3).

In the sensitivity analysis using the stricter Kt/V > 1.5 threshold, female gender (adjusted OR = 3.23, 95% CI 1.70–6.12), government centre treatment (adjusted OR = 7.66, 95% CI 3.62–16.20) and lower dry weight (adjusted OR = 0.97 per kg, 95% CI 0.95–0.99) remained independent predictors, and higher educational attainment additionally emerged as an independent predictor of poorer adequacy (secondary OR = 0.30, 95% CI 0.14–0.67; tertiary OR = 0.27, 95% CI 0.09–0.77). The principal predictors were therefore consistent across both thresholds, whereas the inverse association between education and adequacy was evident only at the stricter cut‐off (Table 3).

Multivariable linear regression analysis was also performed for QoL, demonstrating the SF‐12 physical component summary was independently predicted by younger age (*β* = −0.10 per year, 95% CI −0.19 to −0.02, *p* = 0.019) and shorter dialysis vintage (*β* = −0.21 per year, 95% CI −0.41 to −0.004, *p* = 0.049). Neither centre type nor dialysis adequacy remained an independent predictor of physical QoL after adjustment (*p* = 0.204 and *p* = 0.079, respectively), indicating that the better physical functioning observed at government centres on univariate analysis was largely accounted for by patient age and dialysis vintage. The model explained a modest proportion of the variance (*R* ^2^ = 0.087) (Table 4). For the SF‐12 mental component summary, female gender (*β* = +2.43, 95% CI +0.04 to +4.82, *p* = 0.046) and Malay ethnicity (Chinese versus Malay, *β* = −3.37, 95% CI −6.52 to −0.22, *p* = 0.036) were the only independent predictors, with higher haemoglobin showing a borderline association (*β* = +0.61, *p* = 0.074); the model *R* ^2^ was 0.069 (Table 4).

**TABLE 4:** Multivariable linear regression for predictors of health‐related quality of life (SF‐12 component summaries).

Predictor	SF‐12 physical (PCS)		SF‐12 mental (MCS)	
	Β (95% CI)	*p*	Β (95% CI)	*p*
Age (per year)	−0.10 (−0.19, −0.02)	0.019	0.04 (−0.05, 0.13)	0.377
Female gender	0.38 (−1.84, 2.60)	0.739	2.43 (0.04, 4.82)	0.046
Chinese (vs Malay)	1.14 (−1.79, 4.06)	0.446	−3.37 (−6.52, −0.22)	0.036
Indian/others (vs Malay)	−2.68 (−6.06, 0.70)	0.119	−1.53 (−5.17, 2.11)	0.409
Government centre	1.73 (−0.94, 4.41)	0.204	−0.79 (−3.67, 2.09)	0.591
Haemoglobin	0.21 (−0.41, 0.83)	0.509	0.61 (−0.06, 1.27)	0.074
Dry weight (per kg)	0.04 (−0.04, 0.11)	0.323	0.05 (−0.03, 0.13)	0.250
Good adequacy (Kt/V > 1.2)	2.73 (−0.32, 5.77)	0.079	2.58 (−0.69, 5.86)	0.121
Dialysis vintage (per year)	−0.21 (−0.41, −0.00)	0.049	0.11 (−0.11, 0.34)	0.314
Household income: M40/T20 (vs B40)	0.62 (−1.84, 3.09)	0.619	0.85 (−1.80, 3.50)	0.528
Diabetes mellitus	−1.61 (−3.88, 0.66)	0.165	−0.59 (−3.04, 1.85)	0.633

*Note: β*, unstandardised regression coefficient; SF‐12 PCS model *R* ^2^=0.087; SF‐12 MCS model *R* ^2^ = 0.069; *n* = 294; reference categories: male, Malay, private centre, B40, no diabetes; good adequacy defined as Kt/V > 1.2.

Abbreviations: CI, confidence interval; MCS, mental component summary; PCS, physical component summary.

Dialysis adequacy was not independently associated with either QoL summary score, and continuous Kt/V showed no significant correlation with any KDQOL domain (all *p* > 0.18; SF‐12 physical *r* = −0.02; SF‐12 mental *r* = +0.04). Within this cohort, dialysis adequacy and self‐reported QoL were therefore largely independent.

## 4. Discussion

The most striking finding of this study was that patients at private HD centres were considerably more likely to have poor dialysis adequacy than those receiving treatment at government centres. At an adequacy threshold of Kt/V > 1.2, patients at government centres had approximately 3.6 times the odds of good adequacy after multivariable adjustment, and this association strengthened to more than sevenfold at the stricter Kt/V > 1.5 threshold, confirming that the finding is robust across adequacy definitions. This result runs counter to the prevailing assumption in Malaysia that private facilities, with their perceived advantages in equipment and staffing, deliver better clinical outcomes. International evidence is similarly mixed: While some studies report better adequacy outcomes at private for‐profit centres, a systematic review and meta‐analysis by Dickman et al. found higher mortality odds at for‐profit facilities compared to not‐for‐profit counterparts [17, 18]. One possible explanation for our finding is that government dialysis staff may have greater clinical experience and lower turnover rates than their counterparts in private centres, where high attrition among dialysis technicians is a recognised operational challenge. This hypothesis warrants investigation in future studies.

Benchmarking our cohort against national data provides useful context. The 32nd Report of the Malaysian Dialysis and Transplant Registry (MDTR) reported a mean and median delivered Kt/V of 1.5 for 2024, with 85% of patients nationally achieving a Kt/V of at least 1.2 [16]. Our cohort was closely comparable, with a mean Kt/V of 1.51% and 89% of patients reaching this threshold, indicating that our sample was broadly representative of the national HD population in terms of overall adequacy. When disaggregated by sector, however, a clear divergence emerged: Government centre patients exceeded the national benchmark (mean Kt/V 1.68; 95% achieving Kt/V ≥ 1.2), whereas private centre patients performed at or below it (mean 1.39; 85% achieving Kt/V ≥ 1.2). This reinforces our central finding and illustrates how an aggregate national figure can conceal substantial sector‐level differences.

The MDTR itself reports considerable variation in adequacy between centres, with median delivered Kt/V ranging from 1.0 to 2.0 and the proportion of patients achieving Kt/V 1.2 ranging from 22% to 100%, including centres in which fewer than 30% of patients reached this target [16]. The government–private gap observed in our cohort falls well within this documented national range. Our findings suggested that facility ownership may explain this intercentre variation reported in the MDTR. Notably, the dialysis prescription parameters that most directly determine Kt/V are highly standardised nationally with more than 97% of HD patients receiving 3 sessions weekly, more than 98% being dialysed for at least 4 hours and over 90% being treated at a blood flow rate above 250 mL/min. This uniformity makes it unlikely that gross prescription differences account for the gap we observed, suggesting other patient‐related factors, such as body size, together with unmeasured centre‐related factors, such as vascular access and achieved blood flow that still vary appreciably between centres and were not captured in our current study.

Government centre patients demonstrated better physical functioning and physical component summary scores on the KDQOL‐36. The physical functioning subscale assesses limitations in performing activities ranging from basic self‐care to more vigorous tasks, and low scores in this domain are often driven by fatigue and muscle weakness secondary to uraemia or anaemia. In our cohort, government centre patients were significantly younger, had better haemoglobin levels (10.5 vs 9.99 g/dL, *p* = 0.011), lower iPTH levels and superior dialysis adequacy, suggesting a constellation of factors that together plausibly account for their better physical functioning scores.

Although our introduction framed dialysis adequacy and QoL as closely linked, we found little evidence of an independent relationship between the two in this cross‐sectional cohort. Continuous Kt/V did not correlate with any KDQOL domain, and dialysis adequacy was not an independent predictor of either SF‐12 summary score after adjustment, despite the univariate differences observed between adequacy groups. This apparent decoupling does not diminish the well‐established clinical importance of achieving adequate dialysis, but suggests that, in a population in which the large majority already meet the minimum adequacy target, self‐reported QoL is shaped more strongly by demographic and clinical factors—such as age, gender, ethnicity and dialysis vintage—than by Kt/V itself. The modest explanatory power of our QoL models (*R* ^2^ < 0.10) further underscores that much of the variation in QoL among HD patients remains unexplained by routinely measured clinical parameters, consistent with the multifactorial and partly subjective nature of QoL.

Compared with the 2019 Malaysian validation study by Goh, Lai and Lim, our cohort reported higher scores for burden of kidney disease (45.5 vs 37.5) and effects of kidney disease on daily life (77.0 vs 75.0), though physical and mental component summaries were slightly lower (SF‐12 physical: 38.3 vs 39.8; SF‐12 mental: 50.8 vs 53.1) [11]. Against an older Malaysian cohort reported by Ramatillah et al. in 2017, our patients fared considerably better across most domains including work status, cognitive function, social interaction and sleep [13].

The finding that female patients were more likely to achieve good dialysis adequacy aligns with data from the Malaysian Society of Nephrology and with international literature [19, 20]. This is thought to reflect the smaller body habitus of female patients, which reduces the urea distribution volume and makes it easier to achieve a higher Kt/V for a given dialysis dose. Lower muscle mass, lower physical activity levels and generally better dietary compliance among female HD patients may also contribute, as these factors are associated with lower interdialytic weight gain and more stable predialysis biochemistry [20–22]. The same body size mechanism underlies the association between higher dry weight and poorer adequacy discussed below.

Higher educational attainment showed an unexpected, inverse relationship with adequacy: More highly educated patients were less likely to achieve good Kt/V. This association reached independent significance only at the stricter Kt/V > 1.5 threshold and was attenuated to nonsignificance at the primary Kt/V > 1.2 threshold, indicating that it is sensitive to the definition of adequacy and should be interpreted with caution. The gradient persisted within both government and private centres, so it was not explained by a differential distribution of more‐educated patients across facility types. The basis for this counter‐intuitive finding is not evident from our data and may reflect unmeasured confounding—for example, differences in body size, dietary or fluid adherence, or occupational time pressures affecting session attendance—rather than a direct effect of education, and warrants confirmation in larger prospective studies.

Higher dry weight was independently associated with poorer dialysis adequacy (adjusted OR 0.96 per kg). This is consistent with established dialysis physiology where a greater body mass implies a larger urea distribution volume (V), which lowers Kt/V for a given dialysis dose unless the prescription is scaled accordingly [23]. Our finding suggests that dialysis prescriptions in our cohort may not have been sufficiently individualised to body size, leaving heavier patients less likely to reach the target Kt/V. This interpretation aligns with our observation that female patients, who on average have a smaller body habitus and lower distribution volume, were more likely to achieve good adequacy, reinforcing the central role of body size in determining Kt/V in this population. Future studies incorporating body composition measures, such as lean body mass or bioimpedance spectroscopy, would allow a more refined examination of how body composition, rather than total weight, relates to dialysis adequacy, and would help identify patients in whom dose individualisation is most needed.

Serum calcium and phosphate were each significantly associated with dialysis adequacy on univariate analysis but neither was retained as an independent predictor after multivariable adjustment, suggesting that their associations may be mediated through other variables, such as dry weight or centre type. Higher serum phosphate in particular was associated with poorer adequacy on univariate analysis, in keeping with its role as a marker of inadequate clearance and dietary nonadherence [24]. The clinical importance of mineral bone disorder management in HD patients is nonetheless well established, and monitoring of calcium and phosphate levels remains an essential component of routine HD care [25].

This study has several notable strengths. It appears to be the first in Malaysia to directly compare dialysis adequacy and health‐related QoL between government and private HD centres using a validated, locally adapted instrument, addressing a gap that national registry data, which report only aggregate Kt/V figures, cannot fill. The sample of 294 patients exceeded the prespecified minimum of 274, and recruitment from five geographically distinct centres across Selangor lends reasonable confidence to the stability of the findings. The study population reflects the multi‐ethnic composition of Malaysia, with representation from Malay, Chinese, Indian and other communities across a wide income range, enhancing its contextual validity. The use of the KDQOL‐36, a disease‐specific instrument validated for the Malaysian population [11], ensured that QoL was measured in a culturally meaningful and clinically relevant way. The simultaneous assessment of both sociodemographic and clinical predictors in the multivariate model also provided a more comprehensive picture of the determinants of dialysis adequacy than studies examining either domain in isolation.

Several limitations should be considered when interpreting these findings. First, the cross‐sectional design precludes causal inference, and longitudinal studies are needed to determine whether the observed differences in adequacy and QoL between facility types persist over time. Second, the use of convenience sampling from a single state, and the unequal number of private (*n* = 3) versus government (*n* = 2) centres, may limit the generalisability of findings to the broader Malaysian HD population. Third, the small number of centres means that centre‐level characteristics, such as staffing ratios, dialyser types and institutional protocols, could not be adequately controlled for, and unmeasured centre‐level confounding may have influenced the results. Fourth, income group was based on participants’ self‐identification with the nationally recognised B40/M40/T20 categories rather than on verified household income, and may therefore be subject to some misclassification. Fifth, at the primary Kt/V > 1.2 threshold, the relatively small proportion of patients with poor adequacy limited statistical power for some predictors and widened the corresponding confidence intervals. Sixth, self‐reported KDQOL‐36 data are subject to response bias, and patients’ QoL scores may have been influenced by their clinical state at the time of questionnaire administration. Seventh, several variables known to affect dialysis adequacy, including vascular access type and function, blood flow rates, dialysate flow rates and dialyser membrane characteristics, were not captured in this study. Finally, as multiple KDQOL‐36 subscales were analysed simultaneously, the probability of Type I error is elevated, and borderline significant findings should be interpreted with appropriate caution.

Future research should consider longitudinal study designs, broader multistate sampling and the incorporation of qualitative methods to more thoroughly capture the lived experiences of HD patients across different facility types in Malaysia.

## 5. Conclusion

This study demonstrates that patients receiving HD at government dialysis centres in Malaysia achieve significantly better dialysis adequacy than those at private centres, with government centre patients having approximately three times the odds of good Kt/V after multivariate adjustment. Female gender and government centre affiliation were independent predictors of good dialysis adequacy, whereas higher dry weight was associated with poorer adequacy. QoL was broadly similar between facility types, with the exception of physical functioning and physical component summary scores, which were significantly better among government centre patients. This was likely attributable to their younger age, better haemoglobin levels and superior dialysis adequacy.

These findings challenge the assumption that private dialysis centres in Malaysia deliver equivalent or superior clinical outcomes to government facilities, and underscore the need for regulatory oversight and standardisation of dialysis practice across both sectors. Improving health literacy and patient education particularly for those with lower educational attainment, optimising dialysis prescriptions for individual patient characteristics and addressing the structural factors that contribute to suboptimal adequacy in private centres are priority areas for intervention. Longitudinal studies with broader national representation are needed to confirm these findings and establish the causal pathways linking facility type, patient characteristics and dialysis outcomes in Malaysia’s unique mixed public–private healthcare system.

## Funding

This work was supported by the Universiti Tunku Abdul Rahman Research Fund (No. 6561/1W01).

## Conflicts of Interest

The authors declare no conflicts of interest.

## Data Availability

The data that support the findings of this study are available from the corresponding author upon reasonable request.

## References

[1] Hooi L. S. , Ong L. M. , Ahmad G. et al., A Population-Based Study Measuring the Prevalence of Chronic Kidney Disease Among Adults in West Malaysia, Kidney International. (2013) 84, no. 5, 1034–1040, 10.1038/ki.2013.220. 23760287

[2] Ismail H. , Abdul Manaf M. R. , Abdul Gafor A. H. , Mohamad Zaher Z. M. , and Ibrahim A. I. N. , Economic Burden of ESRD to the Malaysian Health Care System, Kidney International Reports. (2019) 4, no. 9, 1261–1270, 10.1016/j.ekir.2019.05.016. 31517145 PMC6732754

[3] Li P. K. T. , Ng J. K. C. , yan C. G. et al., Navigating the Global Economic Landscape of Dialysis: A Summary of Expert Opinions from the 4th International Congress of Chinese Nephrologists, Kidney Diseases. (2024) 10, no. 5, 384–397, 10.1159/000540152. 39430291 PMC11488833

[4] Sułkowski L. , Matyja A. , and Matyja M. , Social Support and Quality of Life in Hemodialysis Patients: A Comparative Study With Healthy Controls, Medicina. (2024) 60, no. 11, 10.3390/medicina60111732. PMC11596316 39596917

[5] Cohen D. E. , Lee A. , Sibbel S. , Benner D. , Brunelli S. M. , and Tentori F. , Use of the KDQOL-36 for Assessment of Health-Related Quality of Life Among Dialysis Patients in the United States, BMC Nephrology. (2019) 20, no. 1, 10.1186/s12882-019-1295-0. PMC6444438 30935377

[6] Aljawadi M. H. , Babaeer A. A. , Alghamdi A. S. , Alhammad A. M. , Almuqbil M. S. , and Alonazi K. F. , Quality of Life Tools Among Patients on Dialysis: A Systematic Review, Saudi Pharmaceutical Journal. (2024) 32, no. 3, 10.1016/j.jsps.2024.101958. PMC10845059 38322149

[7] Hong W. P. and Lee Y. J. , The Association of Dialysis Adequacy, Body Mass Index, and Mortality Among Hemodialysis Patients, BMC Nephrology. (2019) 20, no. 1, 10.1186/s12882-019-1570-0. PMC6805311 31640580

[8] Bujang Z. , Ramdzan A. R. , Pang N. T. P. , Azzeri A. , Jaafar H. , and Ahmad Mulyadi Lai H. I. , Health-Related Quality of Life Among Hemodialysis Patients and Its Associated Factors: A Scoping Review, Malaysian Journal of Medicine and Health Sciences. (2023) 19, no. SUPP20, 240–249.

[9] Hasan L. M. , Shaheen D. A. H. , El Kannishy G. A. H. , Sayed-Ahmed N. A. H. , and Abd El Wahab A. M. , Is Health-Related Quality of Life Associated With Adequacy of Hemodialysis in Chronic Kidney Disease Patients?, BMC Nephrology. (2021) 22, no. 1, 10.1186/s12882-021-02539-z. PMC8499489 34620098

[10] The National Renal Registry , 31st Report of the Malaysian Dialysis & Transplant Registry 2023, 2023, Kuala Lumpur.

[11] Goh K. K. K. , Lai P. S. M. , and Lim S. K. , Cross Cultural Adaptation and Validation of the Malay Kidney Disease Quality of Life (KDQOL-36), BMC Nephrology. (2019) 20, no. 1, 10.1186/s12882-019-1397-8. PMC6585031 31221116

[12] Hays R. D. , Kallich J. D. , Mapes D. L. , Coons S. J. , and Carter W. B. , Development of the Kidney Disease Quality of Life (KDQOLTM) Instrument, Quality of Life Research. (1994) 3, no. 5, 329–338, 10.1007/BF00451725. 7841967

[13] Ramatillah D. L. , Syed Sulaiman S. A. , Khan A. H. , and Meng O. L. , Quality of Life Among Patients Undergoing Hemodialysis in Penang, Malaysia, Journal of Pharmacy and BioAllied Sciences. (2017) 9, no. 4, 229–238, 10.4103/jpbs.JPBS_191_17. 29456373 PMC5810072

[14] Malaysian Society of Nephrology and Ministry of Health Malaysia , The National Haemodialysis Quality Standards 2018, 2018, Kuala Lumpur.

[15] Jeon J. , Kim G. O. , Kim B. Y. et al., Effects of Kt/V Urea on Outcomes According to Age in Patients on Maintenance Hemodialysis, Clinical Kidney Journal. (2024) 17, no. 5, 10.1093/ckj/sfae116. PMC11099659 38766271

[16] Malaysian Society of Nephrology , 32nd Report of the Malaysian Dialysis and Transplant Registry 2024, 2025.

[17] Dickman S. , Mirza R. , Kandi M. et al., Mortality at For-Profit Versus not-for-Profit Hemodialysis Centers: A Systematic Review and Meta-Analysis, International Journal of Health Services. (2021) 51, no. 3, 371–378, 10.1177/0020731420980682. 33323016

[18] Herrera C. A. , Rada G. , Kuhn-Barrientos L. , and Barrios X. , Does Ownership Matter? An Overview of Systematic Reviews of the Performance of Private for-Profit, Private not-for-Profit and Public Healthcare Providers, PLoS One. (2014) 9, no. 12, 10.1371/journal.pone.0093456. PMC4249790 25437212

[19] Lim Y. and Lim T. , Twelfth Report of the Malaysian Dialysis and Transplant Registry 2004, 2005, Kuala Lumpur.

[20] Miller J. E. , Kovesdy C. P. , Nissenson A. R. et al., Association of Hemodialysis Treatment Time and Dose With Mortality and the Role of Race and Sex, American Journal of Kidney Diseases. (2010) 55, no. 1, 100–112, 10.1053/j.ajkd.2009.08.007. 19853336 PMC2803335

[21] Rezaiee O. , Shahgholian N. , and Shahidi S. , Assessment of Hemodialysis Adequacy and Its Relationship With Individual and Personal Factors, Iranian Journal of Nursing and Midwifery Research. (2016) 21, no. 6, 10.4103/1735-9066.197673. PMC5301063 28194196

[22] Weigert A. , Drozdz M. , Silva F. et al., Influence of Gender and Age on Haemodialysis Practices: A European Multicentre Analysis, Clinical Kidney Journal. (2020) 13, no. 2, 217–224, 10.1093/ckj/sfz069. 32296527 PMC7147302

[23] Vega A. , Ruiz C. , Abad S. et al., Body Composition Affects Urea Distribution Volume Estimated by Watson’s Formula, Journal of Renal Nutrition. (2015) 25, no. 5, 420–425, 10.1053/j.jrn.2015.02.008. 25906704

[24] Copland M. , Komenda P. , Weinhandl E. D. , McCullough P. A. , and Intensive Hemodialysis M. J. A. , Mineral and Bone Disorder, and Phosphate Binder Use, American Journal of Kidney Diseases. (2016) 68, no. 5, S24–S32, 10.1053/j.ajkd.2016.05.024. 27772640

[25] Kim G. H. , Choi B. S. , Cha D. R. et al., Serum Calcium and Phosphorus Levels in Patients Undergoing Maintenance Hemodialysis: A Multicentre Study in Korea, Kidney Res Clin Pract. (2014) 33, no. 1, 52–57, 10.1016/j.krcp.2013.12.003. 26877950 PMC4714160

